# Functional mapping of genotype-environment interactions for soybean growth by a semiparametric approach

**DOI:** 10.1186/1746-4811-6-13

**Published:** 2010-06-02

**Authors:** Qin Li, Zhongwen Huang, Meng Xu, Chenguang Wang, Junyi Gai, Youjun Huang, Xiaoming Pang, Rongling Wu

**Affiliations:** 1 Department of Statistics, University of Florida, Gainesville, FL 32611 USA; 2 Department of Agronomy, Henan Institute of Science and Technology, Xinxiang, Henan 453003, China; 3 National Center for Soybean Improvement, Nanjing Agricultural University, Nanjing, Jiangsu 210095, China; 4 Key Laboratory of Forest Genetics and Tree Engineering, Nanjing Forestry University, Nanjing, Jiangsu 210037, China; 5 School of Forestry and Biotechnology, Zhejiang Forestry University, Lin'an, Zhejiang 311300, China; 6 Center for Computational Biology, Beijing Forestry University, Beijing 100083, China; 7 National Engineering Laboratory for Tree Breeding, Key Laboratory of Genetics and Breeding in Forest Trees and Ornamental Plants, Beijing Forestry University, Beijing 100083, China

## Abstract

**Background:**

Functional mapping is a powerful approach for mapping quantitative trait loci (QTLs) that control biological processes. Functional mapping incorporates mathematical aspects of growth and development into a general QTL mapping framework and has been recently integrated with composite interval mapping to build up a so-called composite functional mapping model, aimed to separate multiple linked QTLs on the same chromosomal region.

**Results:**

This article reports the principle of using composite functional mapping to estimate the effects of QTL-environment interactions on growth trajectories by parametrically modeling the tested QTL in a marker interval and nonparametrically modeling the markers outside the interval as co-factors. With this new model, we can characterize the dynamic patterns of the genetic effects of QTLs governing growth trajectories, estimate the global effects of the underlying QTLs during the course of growth and development, and test the differentiation in the shapes of QTL genotype-specific growth curves between different environments. By analyzing a real example from a soybean genome project, our model detects several QTLs that cause significant genotype-environment interactions for plant height growth processes.

**Conclusions:**

The model provides a basis for deciphering the genetic architecture of trait expression adjusted to different biotic and abiotic environments for any organism.

## Background

In nature, any biological trait of an organism is never isolated from other traits or variables, but rather all of them are integrated under the premise that natural selection has tended to optimize energy absorption and transport within fractal-like distribution networks [1,2]. Such a tendency has led to several universal biological laws; for example, the change of metabolic rate or surface area scales as the 3/4-power of body mass [3,4], organismic growth as a function of age follows a sigmoidal shape [5,6] and metabolic rate increases exponentially with temperature [7]. A number of explicit mathematical equations have been established to describe these biological laws that hold true for all manners of life forms under natural selection.

These mathematical functions have now played an important role in incorporating biological laws into a mapping framework to detect specific quantitative trait loci (QTLs) for growth trajectories and developmental events. R. Wu and group pioneered a series of statistical models, called functional mapping, to map growth and development QTLs through estimating genotype-specific mathematical parameters that define a biological process (reviewed in [8]). In statistics, functional mapping has proven powerful and stable by modeling the patterns of trait development and autocorrelations among different time points measured [9-12]. For some dynamic traits whose expression cannot be mathematically described, nonparametric modeling based on the Legendre polynomial orthogonal has been proposed, thus enhancing the flexibility of functional mapping [13,14]. Functional mapping is genetically relevant, allowing the formulation and test of numerous biologically meaningful hypotheses about the genetic control of growth [15,16]. Some of the most important hypotheses include the timing of a QTL to switch on or off, the duration of its genetic effect, and the pleiotropic effect of this QTL on different aspects of development.

Functional mapping has been further extended to model the genetic control of more complicated biological problems related to QTL-QTL interactions [15,17] and QTL-environment interactions on growth trajectories [10,11]. [15] provided a general procedure within the functional mapping framework for testing the effects of individual genetic components, including the additive, dominance, additive-additive, additive-dominance, dominance-additive and dominance-dominance, on different stages of development. [10,11] incorporated environmental regimes, such as climates or sexes, into functional mapping to explore the consequence of interactions between the QTL and environments in shaping developmental trajectories. Original functional mapping and its extensions were founded on simple interval mapping, whose utilization may be limited in a situation where there are more than one QTL located on a similar region of a chromosome. When such multiple QTL occur, interval mapping-based approaches may find some "ghost" QTLs which provide significant signals for their existence although they do not exist in reality. [18,19] developed a so-called composite interval mapping to separate multiple linked QTLs by integrating the principle of interval mapping for the testing markers and partial regression analysis of all possible other markers as co-factors. Composite interval mapping has been instrumental for the identification of QTLs that are responsible for different traits [20].

The combination of functional mapping and composite interval mapping can be expected to improve the estimation of multiple QTLs for growth trajectories, but this computationally presents a high challenge in terms of the complexity of estimating growth parameters associated with genotypes at each marker as a co-factor. Fortunately, some of these issues have been solved by R. Wu and group [14]. These authors integrate parametrical interval mapping (aimed to test a hypothesized QTL in a test interval) and Legendre polynomial-based nonparametric longitudinal regression analysis (aimed to control the genome background by choosing a proper set of markers as co-factors), facilitating computation, estimation and tests of functional mapping for multiple QTLs. In this article, we extend [14]'s semiparametric idea to illustrate an analytical strategy for mapping QTLs that affect growth trajectories through their main effects or QTL-environment interaction effects. An instructive procedure is described to test the impacts of each of these components on the timing of development and stages of growth in a time course. We report on the detection of QTLs that affect plant height growth trajectories in soybeans by using the approach developed in this article.

## Model

### Experimental Design and Regression Model

Consider a recombinant inbred line (RIL) population in which there are two homozygous genotypes for alternative alleles at each locus. Molecular markers are genotyped for each RIL progeny and analyzed to construct a genetic linkage map used to identify quantitative trait loci (QTLs) that affect growth trajectories. The RIL design allows the same genotype to be replicated in time and space. Assume that there are *n *RILs in a QTL mapping study which are grown in a randomized complete block design in *L *different environments with multiple replicates per environment. Each of the individuals studied is measured for a growth trait at a series of time points, say *T*. We will take means at each time point over replicates in each environment to describe growth trajectories for an RIL. Alternatively, we can use individual plants per RIL to conduct the functional mapping of growth trajectories by modeling the spatial structure of a covariance [21].

Suppose there is a quantitative trait locus (QTL) with genotypes *QQ *(symbolized as 1) and *qq *(symbolized as 2) that controls the dynamic expression of the trait measured. This QTL is located on somewhere on the genetic linkage map constructed. While a pair of markers is used to map a hypothesized QTL on this test interval, *m *markers within a given window length (in cM) are chosen as co-factors to associate with growth trajectories through partial regression analysis. Thus, according to the principle of composite interval mapping [18,19], the phenotypic value of the growth trait, *y*_*il*_*(t)*, for individual *i *measured at time *t *in environment *l*, affected by the putative QTL, is expressed as(1)

where and *μ*_*l*_(*t*) and *α*_*l*_(*t*) are the environment-specific population mean and additive genetic effect for the QTL at time *t*, respectively;  is the indicator variables for individual *i *that specify the QTL genotypes, which is defined as 1 for genotype *QQ *and 0 for genotype *qq*; *α*_*kl*_(*t*) is the environment-dependent additive effect at time *t *associated with marker *k *(except for the interval constructed by the two markers); *x*_*ik *_is the indicator variables that specify the additive effect of marker *k *for individual *i*, respectively, which is defined similarly as ; and *e*_*il *_(*t*) is the environment-dependent residual error at time *t*, normally distributed as . The covariance between the residual errors at different time points *t*_1 _and *t*_2 _within environment *l *is denoted as *σ*_*l*_(*t*_1_,*t*_2_). All the variances and covariances in environment *l *form a (*T *× *T*) covariance matrix **∑**_*l*_.

### Likelihood

Let **y**_*il *_= (*y*_*il*_(1), ⋯, *y*_*il*_(*T*)) be the phenotypic vector at different time points for individual RIL *i *at environment *l*. A mixture model-based likelihood function of the across-replicate henotypic mean vector for the growth trait (**y**) and marker data (**M**) can be written, by assuming that different environments are independent, as(2)

where **Ω **= (*ω*_*j*|*i*_, **Θ**_*jl*_, **Ψ**_*l*_; *j *= 1, 2, *l *= 1, ..., *L*) is a vector of known parameters, that is, the genomic location of the QTL, QTL genotype-specific parameters, and parameters common to all genotypes. In statistics, the location parameter *ω*_*j*|*i *_is the proportion of different mixture normals in equation (2), reflecting the segregation of the QTL in the population, which can be inferred from known marker genotypes at the test interval. For a RIL mapping population, *n *progeny can be classified into four different groups of two-marker genotypes. In each group, the mixture proportion or frequency of a QTL genotype is RIL-specific and can be expressed as the conditional probability of QTL genotype *j *for RIL *i *given its marker genotype [21]. QTL genotype- and environment-specific distribution density, *f*_*jl*_(**y**_*il*_; **Θ**_*jl*_, **Ψ**_*l*_), is assumed to be multivariate normal with expected mean vector **u**_*jl *_= (*u*_*jl*_(1), ⋯, *u*_*jl*_(*T*)) for QTL genotype *j *and covariance matrix **∑**_*l*_.

### Modeling Time-Dependent Genetic Effects

To solve the likelihood function (2), functional mapping models the genotypic means for an assumed QTL at different time points by a biologically meaningful mathematical function. Since a growth trait is considered, the mathematical function used can be a logistic curve, expressed as(3)

where the set of parameters (*a, b, r*) defines the shape of the genotypic curve. Thus, by estimating the three curve parameters, functional mapping can test the differences in geno-typic means over times. Depending on the nature of experimental materials, many other growth equations can also be used [4]. By estimating QTL genotype-specific growth curve parameters for different environments (*a*_*jl*_, *b*_*jl*_, *r*_*jl*_) (*j *= 1, 2; *l *= 1, ..., *L*), the time-dependent additive effect of the QTL in environment *l *is the difference in growth curve between the two genotypes, i.e.(4)

We will use [14]'s nonparametric approach to model time-dependent effects of the markers outside the test interval used as co-factors for partial regression analysis. This approach based on a Legendre polynomial is flexible for curve fitting [22] and has a closed form solution for the marker effects. The Legendre polynomial of order  implemented to model the genetic effects of individual markers is generally expressed as  where

where *s *= 0, 1, ⋯,  and  with min(*t*) and max(*t*) being the first and last time point, respectively.

Combining the parametric model for the QTL effect and the Legendre-based nonparametric model for the marker effects together, we rewrite the time-dependent expected means of different QTL genotypes for RIL *i *in environment *l *as(5)

where  and  are the base population mean vector and the base additive effect vector for marker *k *as a co-factor, respectively.

Combining the QTL and marker effects in composite functional mapping, time-dependent expected means for different QTL genotypes in environment *l *are modelled by  if a logistic curve (3) is considered. The model allows the fitting of any other growth curves.

### Modeling the Covariance Matrix

The covariance structure of serial measurements can be modeled by a number of approaches. One of the commonly used approaches for structuring the covariance is the first order au-toregressive (AR(1)) model [23]. Its advantage lies in the existence of a general expression for calculating the determinant and inverse of the matrix for any number of time points measured. In practice, the assumptions of variance stationarity and correlation stationarity, i.e., the residual variance at different time points is the same, expressed as *σ*^2^, and the correlation between two different time points *t*_1 _and *t*_2 _decreases exponentially in *ρ *with time lag, expressed as , may not hold. [24] used [25] transform-both-sides (TBS) model to meet the first assumption because heteroscedastic variances can be stable after the data are log-transformed. For the AR(1) model, we only need to use  to model the structure of environment-specific covariance matrix **∑**_*l*_. Some other approaches for modeling the covariance structure include the structured antedepedence model [26,27].

### Computing Algorithm

By fixing the position of a hypothesized QTL (in terms of the proportion of recombinant homozygotes) within a test interval, we obtain the maximum likelihood estimates (MLEs) of mean-modeling parameters (**Θ**_*jl*_) and covariance-structuring parameters (**Ψ**_*l*_) with the EM algorithm. In the E step, the posterior probabilities of each QTL genotype for RIL *i *in environment *l *are calculated by

In the *M *step, the log-likelihood equations are derived in terms of Ω_*jl*|*i *_to estimate the growth parameters associated with the QTL effect, basis function parameters associated with the population means and marker effects, and parameters modeling the covariance structure. It is possible to derive the closed forms for the base population means and base additive effects for *m *markers as cofactors, which are expressed as

where

with  and *α*_*l *_= (*α*_*l*_(1), ⋯, *α*_*l*_(*T*)) both for environment *l*.

To estimate the MLEs of the parameters that model the time-dependent QTL effects and covariance matrix, we implement the simplex or Newton-Raphson algorithm in the estimation process with the EM algorithm [28,29]. As shown in [9], the closed forms for estimating the determinant and inverse of the AR(1) covariance matrix can be derived, whose implementation will increase the computational efficiency of functional mapping.

A numerical derivative approach was derived to calculate the sampling errors for the MLEs of the parameters contained within the mixture model (2). The partial derivative of a given function with respect to some parameters *x *and *z *is given by

If *x *and *z *are the same parameters, the above formula simplifies to

where ℓ is the log-likelihood function and *x *and *z *are any parameters of interest in the current model. Taking the inverse of the negative values of the partial derivative matrix, the estimate of the asymptotic sample covariance matrix can be obtained.

### Model Selection

To obtain the best fit of the data, the optimal number of markers involved in the partial regression analysis of composite functional mapping and optimal order of the Legendre polynomial to model the marker effects should be determined. We used the Bayesian information criterion (BIC) [30] as the model selection criterion of the optimal marker number and polynomial order. The BIC is defined as

where  are the MLEs of parameters under the Legendre polynomial of order  dimension,  represents the number of independent parameters under order , and *n *is the total number of observation at a particular time point. The optimal model is one that displays the minimum BIC value. Note that the model selection procedure also incorporates the choice of the best number of markers (flanking a given test interval) as co-factors in composite functional mapping.

## Hypothesis Tests

### Existence of a QTL

Within the framework of functional mapping, a number of biologically meaningful hypotheses can be tested [15]. To understand the genetic architecture of a growth trait, we need to first test the existence of a QTL that affects the dynamic process and shape of the trait. The following hypotheses are formulated to test the genetic control over the entire dynamic process of a trait:(6)

The *H*_0 _states that there are no QTL affecting growth curves (the reduced model), whereas the *H*_1 _proposes that such QTL do exist (the full model). The test statistic for the hypotheses (6) is calculated as the log-likelihood ratio of the reduced to the full model:(7)

where the tildes and hats denote the MLEs of the unknown parameters under the *H*_0 _and *H*_1_, respectively, and **M**_-2 _is the marker information excluding the two tested markers. The critical value of the LR test statistic can be determined by estimating its behavior under the null hypothesis for a whole genome. An empirical approach based on permutation tests by destroying the relationships between the phenotypic values and tested marker interval genotypes [31-33] is usually used to determine the critical threshold of the LR for interval mapping. But this approach cannot be directly used for composite interval mapping in which additional markers (excluding the two tested markers) serve as co-factors to be associated with the phenotypic values. [19] proposed a simulation approach to examine the distribution of the LR values under the null hypothesis. The phenotypic values simulated under the null hypothesis should reflect the effects of the markers as co-factors. This can be done by assuming that the time-dependent phenotypic values in environment ℓ follow a multivariate normal distribution with mean vector

and the covariance matrix with the AR(1) structure. The threshold for the tested interval is estimated as the 5% percentile of the LR values from 1000 simulation replicates. A genome-wide critical threshold is determined by scanning through the entire linkage map, although this process is computationally extensive.

### Pleiotropic Effect of a QTL

If a significant QTL is found, then we can test whether this QTL has a pleiotropic effect on growth trajectories in any two different environments *l*_1 _and *l*_2_. This test is formulated as(8)

for environment *l*_1_, and(9)

for environment *l*_2_. If the null hypotheses above (8 and 9) are both rejected, this implies that the significant QTL detected affects pleiotropically growth trajectories in the two environments. Otherwise, this QTL is operational only in one environment, which causes genotype by environment interactions for growth trajectories. The thresholds for each of the hypotheses (8 and 9) corresponding to two different environments can be determined using the simulation approach as described above.

### QTL by Environment Interaction

In practice, although the QTL is pleiotropic, its effect on growth trajectories may depend on the environment due to QTL by environment interactions. This can be tested for any two environments *l*_1 _and *l*_2 _using(10)

The rejection of the above null hypothesis means that a significant genotype by environment interaction exists due to allelic sensitivity to a varying environment. The log-likelihood ratio test statistics for hypothesis (10) can be determined from simulation studies.

## Results

The model described above was used to analyze a real example from a soybean genome project at Nanjing Agricultural University, China. Two original inbred lines of soybean, Kefeng No. 1 and Nannong 1138-2, as parents were crossed to generate an F_1 _population which was selfed for 7 generations to produce an RIL population composed of two groups of homozygous genotypes each containing two identical alleles from a different parental line. Let 1 and 2 denote the homozygotes derived from the Kefeng No. 1 alleles and Nannong 1138-2 alleles, respectively. A total of 184 RILs were genotyped for 488 molecular markers (restricted fragment length polymorphisms, simple sequence repeats and amplified fragment length polymorphsim) that construct a linkage map with 25 linkage groups covering 4,151.2 cM of the soybean genome [34].

The RILs were planted in a simple lattice design with multiple replicates randomly grown in a plot at the Jiangpu Agricultural Experiment Station of Nanjing Agricultural University, China. The plants were measured for their plant height growth for six to eight times with the first time at the 28th day after emergence and successive seven times every 10 days thereafter. The same study was repeated for year 2005 and 2006. Thus, different years are viewed as two different environments.

**Figure 1:**
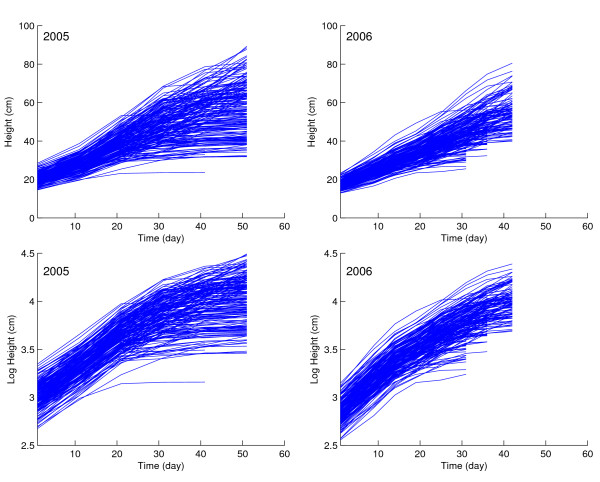
**Growth curves for plant heights in a recombinant inbred lines of soybeans planted in years 2005 and 2006**.

By taking the means for the same RIL over replicates, plant heights are plotted against time in two different years (Fig. 1). The variation among RILs tends to increase with time for raw data (Fig. 1, upper panel), but seems to be constant over time when the data are log-transformed (Fig. 1, lower panel). Thus, we can use AR(1)-based functional mapping to analyze the transformed growth data. Through incorporating the TBS model [24], biological meanings of the growth parameters can be preserved. From Fig. 1, it is seen that plant height growth in each year can well be fit by a logistic curve (3) containing parameters (*a*, *b*, *r*).

**Figure 2:**
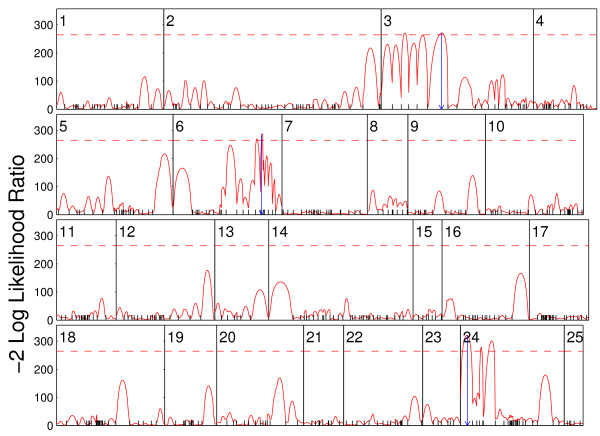
**The profile of the log-likelihood ratios (*LR*) between the full model (there is a QTL) and reduced (there is no QTL) model for plant height growth trajectories across the genome in soybeans planted in years 2005 and 2006**. The genomic position corresponding to the peak of the curve is the maximum likelihood estimate of the QTL localization. The vertical broken lines indicate the positions of markers on this chromosome shown beneath. The map distances (in centiMorgan) between two markers are calculated using the Haldane mapping function. The thresholds for acclaiming the genome-wide existence of a QTL are obtained from 100 permutation tests.

Composite functional mapping will map QTL × year interactions for plant height growth trajectories of soybeans planted in two different years. However, because this approach is computationally very expensive, we will first run traditional functional mapping by [9] and [10,11], aimed to scan growth QTLs throughout the genome. If multiple peaks on the same chromosome are detected from functional mapping, composite functional mapping will be performed to resolve the existence of multiple possible linked QTLs. Log-likelihood ratios from functional mapping were plotted against 25 linkage groups (Fig. 2), from which several peaks were detected on chromosomes 3, 6, and 24. As compared with the critical threshold determined from 100 permutation tests, these peaks were thought to harbor significant QTLs. Yet, these linkage groups have multiple peaks, which is thus subjected to a reanalysis by composite functional mapping.

In composite functional mapping, the growth equation was incorporated to specify the QTL effect, whereas the Legendre polynomial was used to model the marker effects by choosing different numbers of markers as co-factors within a changing window length (in cM) of the test interval of the QTL. The best number of co-factors is determined under different orders of the Legendre polynomial using the BIC criterion (Table 1). The optimal order is found to be 5 for the soybean data. Under these optimal circumstances, only one QTL on each linkage group (3, 6, and 24) was detected by composite functional mapping. The estimates of the locations of these QTLs are given in Table 2, along with the estimates of growth curve parameters for different genotypes at each of the QTL detected (Table 2). It seems that these estimates are reasonably precise because their standard errors estimates are small.

**Table 1:** BIC values under different models of composite functional mapping by choosing markers within different window lengths (in cM) as co-factors. Different orders of the Legendre polynomial are considered under each model.

		Order of Legendre Polynomials			
		
Chromosome	Window Width (cM)	2	3	4	5
3	25	-5833.70	-5815.68	-5925.53	-5941.03
6	10	-7264.87	-7286.02	-7434.39	-7441.61
24	5	-7056.95	-7062.54	-7211.4	-7292.07

**Table 2:** The MLEs of genotypic curve parameters at the two QTLs detected on chromosomes 3, 6, and 24 and sampling errors (in parentheses) of the estimates by composite functional mapping under the optimal order of the Legendre polynomial and the optimal number of co-factors (see Table 1) for plant height growth trajectories of soybeans in two different years (2005 and 2006).

			QTL Genotype					
			
Chromosome	Marker Interval		*QQ*			*qq*		
				
		Year	*a*	*b*	*r*	*a*	*b*	*r*
3	GMKF104b-GMKF177	2005	81.790	66.583	0.131	56.889	47.148	0.133
			(4.013)	(5.148)	(0.006)	(3.283)	(2.520)	(0.007)
		2006	40.314	8.799	0.048	19.148	3.865	0.057
			(3.142)	(1.538)	(0.006)	(3.398)	(1.081)	(0.004)
6	A748V-A397I	2005	82.223	33.853	0.112	54.908	22.763	0.115
			(10.290)	(1.364)	(0.001)	(4.534)	(1.422)	(0.002)
		2006	56.924	6.072	0.044	26.879	2.460	0.055
			(3.711)	(0.417)	(0.001)	(2.540)	(0.193)	(0.003)
24	sat_231- LE23T	2005	62.894	46.764	0.124	100.571	72.397	0.122
			(7.006)	(9.020)	(0.006)	(10.245)	(13.981)	(0.006)
		2006	69.695	223.809	0.175	101.781	308.94	0.174
			(9.148)	(52.231)	(0.010)	(12.711)	(69.850)	(0.010)

**Figure 3:**
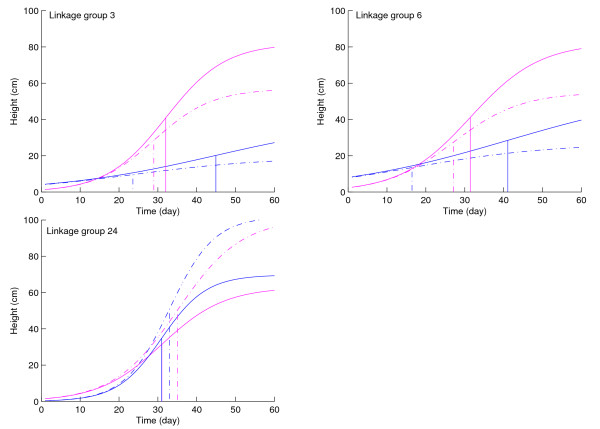
**Growth curves of two genotypes at each of the QTLs detected on linkage groups 3, 6 and 24 for years 2005 (purple) and 2006 (blue)**. For each year, the solid and dot curves correspond to a genotype composed of alleles from parents Kefeng No. 1 and Nannong 11382, respectively. The times at the inflection point of a growth curve are shown by vertical lines.

Figure 3 illustrates the growth curves of QTL genotypes in years 2005 and 2006 drawn with the estimates of curve parameters in Table 2. All the QTLs detected display increasing additive effects with time. At the QTLs on linkage groups 3 and 6, parent Kefeng No. 1 contributes favorable alleles to increasing plant growth, whereas such favorable alleles are derived from parent Nannong 1138-2 at the QTL on linkage group 24. After significant QTLs were detected, hypothesis tests (8 and 9) were used to investigate whether these QTLs trigger pleiotropic effects on growth trajectories for soybeans in different years. At each of the detected QTLs, the null hypotheses of both (8 and 9) are rejected, which suggests that they are all pleiotropic QTLs affecting growth trajectories in both years. A further test with hypothesis (10) suggests that, although two of these QTLs (on chromosomes 3 and 6) are pleiotropic, the magnitude and temporal pattern of their additive genetic effects on growth traits vary from year to year (see Fig. 3). Such interactions between the QTLs and years determine year-specific differences in growth trajectories of plant heights in soybeans. The QTL on chromosome 24 affect plant growth trajectories in a consistent pattern over years, displaying no genotype × year interactions.

By estimating and testing the inflection point of a logistic curve using ln *b*/*r*, we further investigate how the detected QTLs exert significant impacts on the timing of maximum growth rate (Fig. 3). The inflection point of growth curves in 2005 displays a difference of three days between the two genotypes (32 vs. 29) at the QTL on chromosome 3, whereas this difference in 2006 is as large as 21 days (45 vs. 24). For the QTL on chromosome 6, we detect a similar pattern for the difference in the inflection point, i.e., 4 days (31 vs. 27) in 2005 and 25 days (41 vs. 16) in 2006. Yet, unlike these two QTLs, there is no year-specific difference in the inflection point for the QTL on chromosome 24, which does not exhibit a significant effect on this developmental characteristic in both years. Although favorable alleles that increase plant growth are contributed by parent Kefeng No. 1 for QTLs detected on chromosomes 3 and 6, parent Nannong 1138-2 contributes such a favorable allele for the QTL on chromosome 24 (Fig. 3).

## Discussion

Genetic mapping has proven to be a powerful approach for map individual genes or quantitative trait loci (QTLs) that control a quantitatively inherited trait [35]. However, this approach would not gain too much insight into the genetic control mechanisms for phenotypic variation if some statistical and biological issues related to the approach are not resolved. Zeng and others are among the first who systematically investigate the effects of multiple linked QTLs on the power of genetic mapping, and further proposed so-called composite interval mapping to separate linked QTLs through using the markers outside the test interval to make the background control of the genome [18,19,36]. Many other approaches that take into account a specific statistical, computational or genetic issue of genetic mapping have been developed [37-43]. All these approaches have been instrumental for the characterization of QTLs that control quantitative traits of interest to agriculture, biology and health sciences [44,20,46].

More recently, genetic mapping has been integrated with some fundamental biological principles, aimed to generate biologically more meaningful discoveries related to trait formation and development. One of the most important products for this integration is the formulation of a series of statistical models, called functional mapping [9,15,16,24,8]. Functional mapping capitalizes on the mathematical aspects of biological processes to model the temporal pattern of genetic effects exerted by a QTL in time course. It offers tremendous advantages in the generation of testable biological hypotheses and synthesis of different disciplines for a more comprehensive understanding of biology. Functional mapping have now been extended to explore the roles gene-gene, gene-environment, gene-sex interactions play in directing growth trajectories of a complex trait [15,10,11]. Combined with composite interval mapping, functional mapping has been shown to have more power to separate linked QTLs on the same chromosome [14]. This composite functional mapping uses a parametric approach to model the temporal effect of the QTL effect and a nonparametric approach based on the Lengendre polynomial [22,47,13] to model the temporal effects of different markers as co-factors. Parametric modeling preserves the biological relevance of the original functional mapping, whereas nonparametric modeling increases the flexibility of functional mapping and its computational efficiency.

In this report, we incorporate composite functional mapping to map QTLs that interact with environments to regulate the process of trait development. While traditional functional mapping detected significant signals for the existence of QTLs for plant height growth trajectories in a recombinant inbred line (RIL) population of soybeans, the new composite functional mapping shows great power to separate multiple linked QTLs on the same chromosome. In total, we detected three significant QTLs on chromosomes 3, 6 and 24 that affect height growth in different years. These QTLs were also found by an allometric model [29]. By integrating environmental factors, the new model detected these QTLs to be operational in both years. However, the magnitudes of their effects vary between different years, showing significant QTL by year interactions. It seems that plant height growth is under strong genetic control. In the functional mapping of rice, [27] found several significant QTLs for plant heights in two contrasting climates although the expression of these QTLs is not environment-dependent.

Currently, there are three different hypotheses proposed to explain the mechanisms of genotype × environment interactions-heterozygosity, allelic sensitivity, and gene regulation [48,49]. The new model provides a genome-wide search for QTLs that act in terms of the second hypothesis, i.e., the environment-dependent change of a trait is caused by differentiate expression of a QTL in different environments. However, it is possible to modify the model to test the other hypotheses. For example, the gene regulation hypothesis can be tested by assuming different but epistatically interacting QTLs for mean growth curves and individual growth curves across different environments [50,51]. Our approach combines powerful statistics and molecular genetics with developmental and ecological mechanisms underlying biological features, relationships and processes to shed light on the genetic basis of complex traits. Such a *mechanistic *strategy will be powerful to address fundamental questions about plant development and plastic response to changing environments.

## Competing interests

The authors declare that they have no competing interests.

## Authors' contributions

QL, CW, and YH formulated the model and performed simulation studies and data analysis, ZH and JG designed and performed the experimental work. MX and XP provided biological inputs. JG and RW coordinated the work. RW conceived the model of functional mapping and wrote the manuscript. All authors read and approved the final manuscript.
